# Circulating neurotrophins and hemostatic risk factors of atherothrombotic cardiovascular disease at baseline and during sympathetic challenge: the SABPA study

**DOI:** 10.1038/s41598-021-81946-6

**Published:** 2021-01-27

**Authors:** Roland von Känel, Mark Hamer, Annemarie Wentzel, Leoné Malan

**Affiliations:** 1 Department of Consultation-Liaison Psychiatry and Psychosomatic Medicine, University Hospital Zurich, University of Zurich, Culmannstrasse 8, 8091 Zurich, Switzerland; 2 Hypertension in Africa Research Team (HART), North-West University, Potchefstroom, South Africa; 3 Division of Surgery and Interventional Science, Faculty of Medical Sciences, University College London, London, UK

**Keywords:** Cardiovascular diseases, Predictive markers, Risk factors, Glial biology, Neurotrophic factors, Stress and resilience

## Abstract

Sympathetic activation may trigger acute coronary syndromes. We examined the relation between circulating neurotrophic factors and hemostatic risk factors of atherothrombotic cardiovascular disease at baseline and in response to acute mental stress to establish a brain–heart link. In 409 black and white South Africans, brain-derived neurotrophic factor (BDNF) and fibrinolytic measures were assessed at baseline. Glial cell-derived neurotrophic factor (GDNF), S100 calcium-binding protein (S100B), von Willebrand factor (VWF), fibrinogen and D-dimer were assessed at baseline and 10 min after the Stroop test. Neurotrophins were regressed on hemostatic measures adjusting for demographics, comorbidities, cardiometabolic factors and health behaviors. Higher baseline BDNF was associated with greater stress-induced increase in fibrinogen (*p* = 0.003) and lower D-dimer increase (*p* = 0.016). Higher baseline S100B was significantly associated with higher baseline VWF (*p* = 0.031) and lower fibrinogen increase (*p* = 0.048). Lower baseline GDNF was associated with higher baseline VWF (*p* = 0.035) but lower VWF increase (*p* = 0.001). Greater GDNF (*p* = 0.006) and S100B (*p* = 0.042) increases were associated with lower VWF increase. All associations showed small-to-moderate effect sizes. Neurotrophins and fibrinolytic factors showed no significant associations. The findings support the existence of a peripheral neurothrophin-hemostasis interaction of small-to-moderate clinical relevance. The implications for atherothrombotic cardiovascular disease need further exploration.

## Introduction

Fueling the scientific bases of a brain–heart link, there is accumulating evidence for a role of circulating neurotrophic factors (a.k.a. neurotrophins), such as brain-derived neurotrophic factor (BDNF) and S100 calcium-binding protein B (S100B), in atherothrombotic cardiovascular disease (CVD). For instance, low serum levels of BDNF were found in patients with stable coronary heart disease (CHD) versus non-CHD controls^1^ and BDNF was associated with incident CHD in the Framingham study^2^. Compared to those with stable CHD, patients with unstable CHD showed elevated BDNF levels in the coronary circulation and greater expression of BDNF in coronary arteries, suggesting that BDNF is critically involved in atherogenesis and plaque instability^3^. Serum levels of S100B, indicating neuroinflammation and increased blood brain barrier permeability^4^, were higher in patients with acute coronary syndrome (ACS) than in patients with stable CHD^5^. Moreover, serum S100B levels showed direct correlations with cardiac enzyme levels in ACS patients and with infarct size in a rat model of cardiac ischemia–reperfusion injury^5^. Another emerging neurotrophin is glial cell-derived neurotrophic factor (GDNF) with lower levels possibly being associated with metabolic factors relevant to CVD risk^6^.

Alterations in the hemostatic system with respect to a hypercoagulable state play a pivotal role in the initiation, progression and clinical manifestation of atherothrombotic diseases^7–9^. Hemostatic factors such as elevated plasma levels of von Willebrand factor (VWF), fibrinogen, fibrin D-dimer and plasminogen activator inhibitor (PAI)-1 have been associated with an increased risk of CHD, independent of traditional risk factors of CVD^10–12^. Although it is increasingly acknowledged that the brain and the hemostatic system do not function in isolation from each other^13^, research on associations between neurotrophins and hemostatic factors in the human circulation is still in its infancy. Such studies could help to elucidate mechanisms involved in the brain–heart link relevant to atherosclerosis. Two previous studies in patients with CHD showed inverse associations of BDNF with fibrinogen^14^ and VWF^2^. The latter suggests a link between BDNF and VWF in endothelial dysfunction, a key mechanism in atherosclerosis. Indeed, BDNF promotes vascular endothelial cell integrity but it is also directly released from endothelial cells^15^ and VWF is a marker of endothelial cell injury^16^. BDNF is also required for intramyocardial vessel stabilization, which are compromised in CHD and ACS^15^. Similarly, it has been suggested that circulating S100B could contribute to endothelial cell dysfunction, based on a positive association between S100B and VWF levels observed in patients with multiple trauma^17^. In vitro, lower levels of recombinant human BDNF were correlated with more rapid clotting and longer clot lysis, resulting in the formation of larger thrombi^18^.

The pathophysiology of ACS triggering, for instance through strenuous exercise and emotional stress^19^, involves sympathetically induced activation of the coagulation system^20^. The VWF, mediating platelet adhesion and aggregation in ACS^21^, fibrinogen and D-dimer, indicating fibrin formation, although not PAI-1, have been shown to be acute stress-responsive hemostatic factors^22^. Circulating levels of BDNF and S100B were also found to be responsive to acute mental stress in humans^23^ and to restraint stress in rats^24^, respectively. Studies on whether circulating levels of neurotrophins, both at baseline and during stress, are associated with stress-induced hypercoagulability could provide new insight into the pathophysiology of the brain–heart link in ACS.

The primary aim of this study was to investigate the association between circulating levels of BDNF, S100B and GDNF with hemostatic factors reflecting endothelial function/integrity, coagulation activation, and fibrinolysis both at baseline and during sympathetic challenge in a cohort of urban black and white teachers from South Africa. In this cohort, we previously reported that compared to whites, blacks had lower baseline BDNF levels^25^, higher hemostatic factors indicative of a baseline-procoagulant state^26^ and lower hemostatic stress reactivity^27^. Therefore, and as cardiovascular burden is projected to rapidly increase in black Africans from sub-Saharan urban regions^28^, a secondary aim of this study was to examine whether the relationship between neurotrophic and hemostatic measures would differ between blacks and whites at baseline and in response to mental stress.

We hypothesized that BDNF levels (measured at baseline only) would show an inverse association with hemostatic factors of a hypercoagulable state at baseline and in response to stress, adjusting for important demographic and health characteristics known to affect hemostasis. However, due to the scant available literature, we considered all other analyses exploratory regarding the direction of a relationship between neurotrophic and hemostatic factors and the role of ethnicity therein.

## Methods

### Participants

Study participants were recruited as part of the Sympathetic Activity and Ambulatory Blood Pressure in Africans (SABPA) study between 2/2008 and 5/2009^29^. The study protocol, conducted in accordance with the Declaration of Helsinki, was approved by the Ethics Review Board of the North-West University, Potchefstroom Campus. All participants provided written informed consent. The SABPA study had a target sample of 409 teachers to assure similar socio-economic class, aged 25–65 years, and working in the Dr Kenneth Kaunda Education district in the North West Province, South Africa. To increase generalizability of study findings, we analysed the data of the entire cohort, but made statistical adjustment for health characteristics as potentially important confounders.

### Procedures

On the same day, Monday through Thursday, all participants were equipped with a 24-h ambulatory BP measurement device between 7:00 and 8:00 am at their respective schools and admitted to the Metabolic Unit Research Facility of the North-West University at 5:00 pm. They completed questionnaires for an assessment of demographic data, general health and health behaviors. A standardized dinner was served at 6:30 pm. A last snack with beverages was provided at 8:30 pm. Afterwards participants engaged in recreational activities before going to bed at 10:00 pm. They were woken at 5:45 am. Anthropometric measurements were obtained, the ambulatory BP monitor was removed, and fasting blood samples were collected to determine routine laboratory parameters and neurotrophic factors. A sterile winged infusion set was left in situ with a heparin block to prevent clotting. After a standardized breakfast, participants underwent a standardized laboratory stress procedure.

### Stress protocol

Participants were administered the Stroop Color-Word Conflict test to induce mental stress, which provokes reproducible cardiovascular reactivity^30^ and hemostatic activation^31^. For one minute, words on a cardboard describing a specific color, but written in different colors, were shown to participants. They were asked to verbally indicate the ink color of a given word but not to read the color represented by the word. In order to increase the stress/ challenge level, participants were asked to progress as fast as possible and to correct answers that were wrong; they received a monetary incentive according to their performance on completion of the task. To determine cardiovascular reactivity, beat-to-beat BP and heart rate were recorded with the Finometer device at baseline and during stress (Finapres Medical Systems, Amsterdam, the Netherlands). For this purpose, the average of the recordings of the last minute of the 5-min baseline phase and of the last 20 s of the 1-min stress phase was calculated. To determine hemostatic reactivity, citrated blood samples to assay hemostatic factors were collected immediately before the Stroop test and 10 min thereafter.

### Measures

#### Neurotrophic factors

Serum BDNF was analyzed with a Quantikine Colorometric Sandwich Immunoassay (R&D Systems, USA & Canada; catalogue number: DBD00) with intra‐ and inter-assay coefficients of variation (CV) < 7% and 12%, respectively. Serum GDNF was determined with the Human GDNF DuoSet ELISA kit (R&D Systems, USA & Canada; catalogue number DY212) with intra- and inter-assay CV < 9%. Serum S100B was measured with an electrochemiluminescence immunoassay on the automated Cobas e411 (Roche, Basel, Switzerland) with intra- and inter-assay CV < 5%. All samples were analyzed in duplicates.

#### Hemostatic factors

Fibrinogen was determined with a viscosity-based method and D-dimer with the STA-Liatest D-di immunoturbidimetric assay (STA Compact, STAGO Diagnostic, Roche, Asnières, France). ELISA kits were used to quantify PAI-1(TriniLIZE PAI-1 Antigen, Trinity Biotech, Bray, Ireland) and VWF antigen (antibodies from DAKO, Gauteng, South Africa). Clot lysis time (CLT) was measured with a turbidimetric method^32^. Intra- and inter-assay CV were < 10% for all hemostatic assays. All samples were analyzed in duplicates.

#### Covariates

Collection of self-reported data included information on ethnicity, sex, age and history of stroke and myocardial infarction. Human immunodeficiency virus (HIV) infection was investigated with a rapid antibody test in plasma (First Response Kit; PMC Medical, Daman, India) and confirmed with the Pareekshak test (BHAT Bio-Tech, Bangalore, India). With participants in their underwear, height and weight were measured to the nearest 0.1 cm and 0.1 kg to calculate the BMI. Hemoglobin A1c (HbA1c) was determined from EDTA whole blood samples with a turbidimetric inhibition immunoassay using the Roche Integra 400 (Roche, Basel, Switzerland). High-sensitivity C-reactive protein (CRP), γ-glutamyl transferase (GGT), total cholesterol (T-C) and high-density lipoprotein cholesterol (HDL-C), were measured in serum with two sequential multiple analyzers (Konelab 20i, Thermo Scientific, Vantaa, Finland; Unicel DXC 800, Beckman and Coulter, Munich, Germany). A categorical variable with CRP ≤ 3 mg/L, 3–10 mg/L and > 10 mg/L, indicating different CVD risk, was formed and used as a covariate. Levels of GGT were categorized as < 41 IU/L vs. ≥ 41 IU/L as a marker of chronic excessive alcohol consumption^33^. As an index of dyslipidemia, the T-C/HDL-C ratio was used as a covariate. Ambulatory BP was monitored with the Cardiotens apparatus (CE0120, Meditech, Budapest, Hungary) over 24 h with 30-min intervals during the day (8:00 am to 10 pm) and 60-min intervals during the night (89% mean successful inflation rate). Mean 24-h arterial pressure, an integrative measure of systolic and diastolic BP, was calculated with the formula [2/3(diastolic BP) + 1/3 (systolic BP)] and used as a covariate. Estimated glomerular filtration rate (eGFR) was computed with the 4-variable MDRD formula considering serum creatinine, age, ethnicity and sex^34^. Current smoking was determined based on self-report and/or serum cotinine levels analyzed by an accredited pathology lab (AMPATH, Pretoria, Gauteng, South Africa) with the Modular Roche Automated Analyser (Basel, Switzerland). Actical accelerometers (Montréal, Québec, Canada) worn over a 7 d monitoring period were used to form a categorical variable of light, moderate or vigorous physical activity^35^.

### Statistical analysis

Data were analyzed using SPSS version 25 (SPSS, Chicago, IL) with level of significance set at *p* < 0.05 (two-tailed). We did not adjust *p*-values for multiple comparisons, but show effects sizes of relationships. Reasons for this were a) the exploratory nature of our study in a nascent research area bearing the risk of deeming truly important relationships non-significant, and b) indication of the same biological process (i.e. hemostatic function) of outcome variables^36,37^. Effect sizes were expressed as Cohen’s d with 0.2, 0.5, and 0.8 indicating small, medium and large effects, respectively^38^. We also report on main effects with borderline significance (*p* < 0.10) to show trends that may additionally help pattern recognition of a brain-hemostasis cross-talk. Due to skewed distributions, values of neurotrophins and hemostatic factors were log transformed before the analyses. Several variables had missing values (< 5% for each of these variables) which were replaced with the expectation maximization algorithm (see supplementary information for numbers of missing values). Log baseline values, log post-stress values and stress-induced percent changes in neurotrophins and hemostatic factors that were three standard deviations above or below the sample mean were considered outliers (see supplementary information for number of outliers). The participants with these outliers were excluded in correlation analyses, repeated measure analyses of variance and regression models. For these analyses with D-dimer as an outcome, two participants on oral anticoagulants, lowering D-dimer levels, were also excluded.

Characteristics of black and white South Africans were compared with t-tests for continuous variables and with chi‐square tests for categorical variables. Pearson correlations were used to analyze the strength of the relationship between neurotrophins and hemostatic factors. Stress reactivity of cardiovascular parameters, hemostatic and neurotrophic factors was tested with repeated measure analysis of variance. Hierarchical regression analysis, using forced entry for blocks of covariates, was employed to identify whether neurotrophic measures would significantly be linked with hemostasis measures, independent of covariates, both at baseline and during acute mental stress. For the latter analysis, stress-induced changes in neurotrophins and hemostatic factors were calculated as percent change from baseline values. Covariates in each block of the regression models were selected a priori, based on the literature, as they might potentially confound associations with hemostatic factors: (a) ethnicity; (b) demographic factors: sex and age; (c) comorbidities: HIV positive status and atherothrombotic disease history; (d) metabolic factors: BMI, HbA1c, T-C/HDL-C, mean 24-h arterial pressure, eGFR and CRP categories; (e) health behaviors: current smoking, physical activity and excessive alcohol consumption. Bar graphs across quartiles of either baseline values or stress-induced percent changes in neutrophins were drawn to illustrate associations of neurotrophins that were found to be significant in fully adjusted models. As a complementary analysis, we additionally entered interaction terms between ethnicity and neurotrophic measures to explore whether the relationship between neurotrophic and hemostatic measures would significantly differ in black from white South Africans. Regression outputs were verified for the absence of multicollinearity and influential outliers in the set of predictor variables with Cook’s distance.

## Results

### Participant characteristics

Table 1 shows that with the exception of the distributions of sex, age and atherothrombotic diseases, all other variables were significantly different between blacks and whites. Blacks had a worse CVD risk profile in terms of most metabolic factors and adverse health behaviors. Relative hypercoagulability was evident in blacks with higher levels of hemostatic factors indicating prothrombotic activity and lower levels of measures indicating fibrinolytic activity. In terms of neurotrophins, BDNF and GDNF levels were lower and S100B level was higher in blacks than whites.

**Table 1:** Characteristics of all study participants and compared between black and white South Africans.

Variables	All (n = 409)	Blacks (n = 200)	Whites (n = 209)	*p* (groups)
Female sex, n	207 (50.6)	99 (49.5)	108 (51.7)	0.660
Age, years	45 (38.0–52.0)	44 (28.0–51.0)	47 (38.5–53.0)	0.495
Human immunodeficiency virus positive status, n	19 (4.6)	19 (9.5)	0 (0)	< 0.001
Atherothrombotic disease, n	5 (1.2)	2 (1.0)	3 (1.4)	1.000
Body mass index, kg/m^2^	27.7 (24.2–32.0)	29.8 (24.9–34.0)	26.9 (23.6–30.3)	< 0.001
Hemoglobin A1c, %	5.60 (5.30–5.90)	5.76 (5.50–6.20)	5.40 (5.20–5.70)	< 0.001
Total-cholesterol / high-density lipoprotein-cholesterol, ratio	4.33 (3.49–5.76)	4.03 (3.26–5.10)	4.74 (3.64–6.24)	0.005
Mean 24-h arterial pressure, mmHg	94.7 (88.3–101.3)	98.8 (90.7–107.7)	91.7 (86.2–97.0)	< 0.001
Estimated glomerular filtration rate, mL/min/1.73m^2^	101. 4 (86.5–116.7)	112.6 (93.5–129.7)	94.0 (83.2–106.0)	< 0.001
**High-sensitivity C-reactive protein**				
≤ 3 mg/L, n	218 (53.3)	69 (34.5)	149 (72.0)	< 0.001
3–10 mg/L, n	136 (33.3)	85 (42.5)	51 (24.6)	
> 10 mg/L, n	55 (13.4)	46 (23.0)	9 (4.4)	
Current smokers, n	75 (18.3)	50 (25.0)	25 (12.1)	0.001
**Physical activity, intensity**				
Light, n	252 (61.6)	147 (73.5)	105 (50.7)	< 0.001
Moderate, n	120 (29.3)	42 (21.0)	78 (37.7)	
Vigorous, n	37 (9.0)	11 (5.5)	26 (12.6)	
Excessive alcohol consumption, n	131 (32.0)	102 (51.0)	29 (14.0)	< 0.001
Brain-derived neurotrophic factor, ng/mL	1.45 (1.07–1.84)	1.33 (0.91–1.79)	1.53 (1.17–1.87)	< 0.001
Glia cell-line derived neurotrophic factor, pg/mL	25.2 (17.2–92.7)	21.3 (16.5–44.2)	37.0 (18.7–243)	< 0.001
S100 calcium-binding protein, µg/L	0.041 (0.031–0.058)	0.050 (0.036–0.069)	0.038 (0.029–0.046)	< 0.001
von Willebrand factor, %	72.6 (59.3–91.5)	88.8 (76.9–108.0)	60.9 (54.1–70.1)	< 0.001
Fibrinogen, g/L	3.16 (2.77–3.67)	3.45 (2.89–3.98)	2.97 (2.66–3.44)	< 0.001
D-dimer, ng/mL	260 (167–410)	305 (183–458)	230 (134–324)	< 0.001
Plasminogen activator inhibitor-1, ng/mL	27.2 (20.4–35.4)	35.2 (27.1–42.3)	21.3 (16.0–27.3)	< 0.001
Clot lysis time, min	77.7 (69.6–86.0)	80.5 (73.0–82.8)	74.7 (68.2–82.8)	< 0.001

Values are percentages or median with inter-quartile range.

### Neurotrophins and hemostatic factors at baseline

Several significant correlations emerged between baseline levels of the five hemostatic factors (all *p*-values < 0.001). There were direct associations of VWF with fibrinogen (r = 0.27), D-dimer (r = 0.25), PAI-1 (r = 0.40) and CLT (r = 0.20); of fibrinogen with D-dimer (r = 0.28), PAI-1 (r = 0.17) and CLT (r = 0.20); and of PAI-1 with CLT (r = 0.43). As per associations between baseline levels of neurotrophins, there was an inverse correlation between GDNF and S100B levels (r = − 0.19, *p* < 0.001); BDNF levels showed no significant correlation with GDNF and S100B levels.

**Figure 1:**
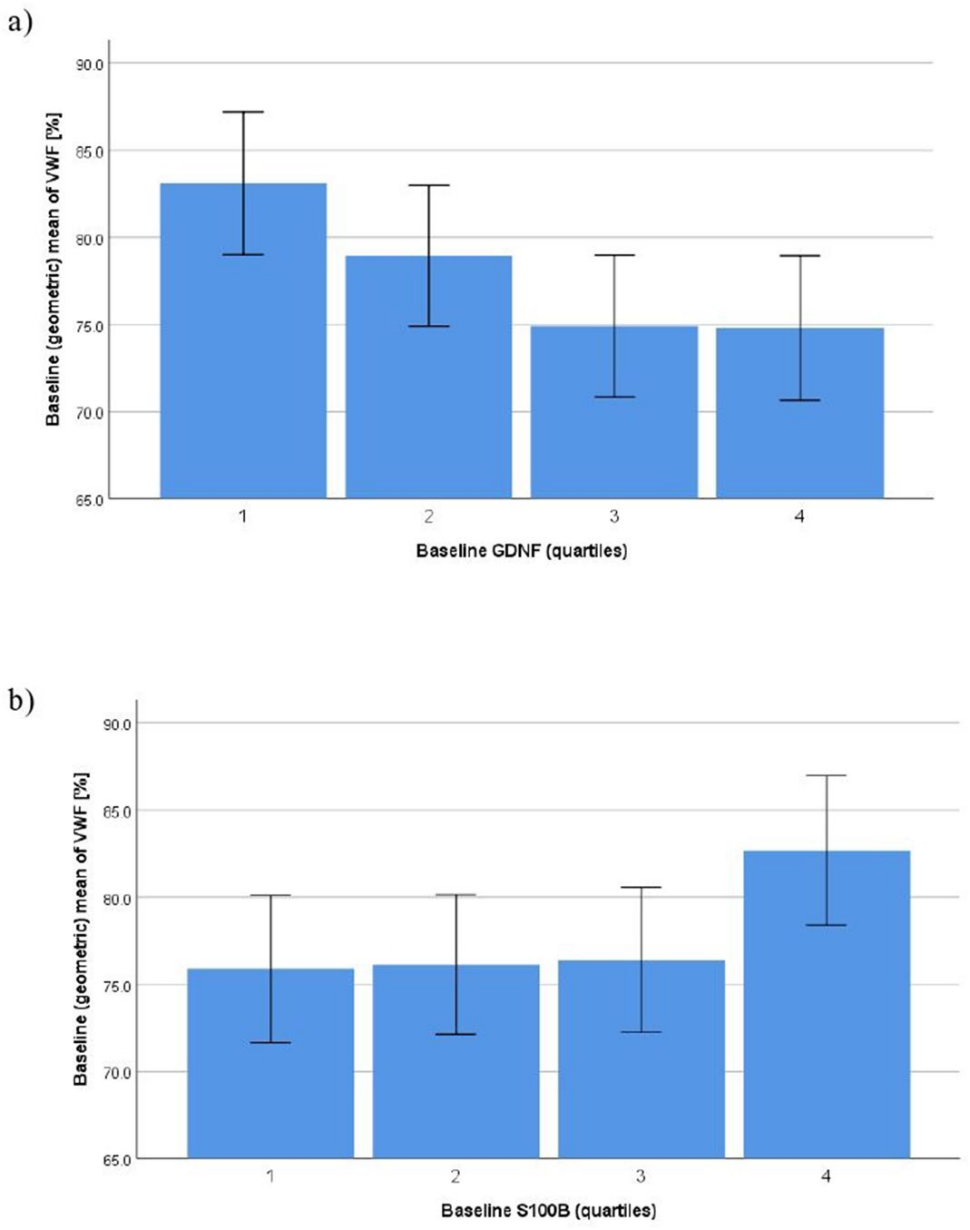
Fully adjusted significant associations between baseline values of neurotrophins across quartiles (1 = lowest quartile, 4 = uppermost quartile) and stress-induced percent changes in von Willebrand factor (VWF). GDNF, glial cell-derived neurotrophic factor; S100B, S100 calcium-binding protein.

The regressions of baseline values of neurotrophins on hemostatic factors, subsequently adjusted for blocks of covariates, are summarized in Table 2. Fully adjusted models showed significance for VWF, which was inversely associated with GDNF (*p* = 0.035, d = 0.21) (Fig. 1a) and positively associated with S100B (*p* = 0.031, d = 0.22) (Fig. 1b). Also in the fully adjusted model, a trend towards significance was seen for longer CLT with higher S100B levels (*p* < 0.06, d = 0.19). Ethnicity-adjusted significant associations of BDNF with fibrinogen (*p* = 0.001, d = 0.35), D-dimer (*p* = 0.035, d = 0.21) and PAI-1 (*p* = 0.040, d = 0.20) became non-significant with further adjustment for sex and age. The unadjusted significant and direct associations of S100B with fibrinogen (*p* = 0.001, d = 0.33), D-dimer (*p* = 0.041, d = 0.20), PAI-1 (*p* < 0.001, d = 0.47) and CLT (*p* = 0.046, d = 0.20) became non-significant with adjustment for ethnicity. Likewise, the unadjusted significant and inverse association of GDNF with PAI-1 (*p* = 0.020, d = 0.24) and of GDNF with CLT (*p* < 0.07, d = 0.19), showing borderline significance, became both non-significant with adjustment for ethnicity. In complementary analyses, adjusting for all covariates, all interaction terms between ethnicity and neurotrophic measures turned out to be non-significant (data not shown).

**Table 2:** Regression models for the associations between neurotrophins and hemostatic factors at baseline.

Variables entered	Log VWF [%]	Log fibrinogen [g/L]	Log D-dimer [ng/mL]	Log PAI-1 [ng/mL]	Log CLT [min]
**Log BDNF [ng/mL]**	− 0.038 (− 0.101, 0.024)	0.047 (0.003, 0.091)*	0.104 (− 0.055, 0.264)	− 0.162 (− 0.239, − 0.085)***	− 0.032 (− 0.069, 0.004)(*)
+ ethnicity	0.037 (− 0.015, 0.088)	0.075 (0.032, 0.117)***	0.171 (0.012, 0.330)*	− 0.065 (− 0.127, − 0.003)*	− 0.017 (− 0.053, 0.020)
+ sex, age	0.022 (− 0.032, 0.077)	0.030 (− 0.013, 0.072)	0.086 (− 0.080, 0.252)	− 0.025 (− 0.090, 0.039)	0.004 (− 0.033, 0.042)
+ comorbidities	0.025 (− 0.030, 0.079)	0.028 (− 0.014, 0.070)	0.082 (− 0.084, 0.248)	− 0.024 (− 0.089, 0.041)	0.004 (− 0.034, 0.042)
+ metabolic factors	0.019 (− 0.035, 0.073)	0.010 (− 0.026, 0.046)	0.062 (− 0.100, 0.224)	− 0.032 (− 0.096, 0.032)	− 0.009 (− 0.041, 0.023)
+ health behaviors	0.016 (− 0.038, 0.070)	0.011 (− 0.024, 0.046)	0.055 (− 0.108, 0.217)	− 0.031 (− 0.095, 0.033)	− 0.005 (− 0.037, 0.026)
**Log GDNF [pg/mL]**	− 0.037 (− 0.054, − 0.019)***	− 0.006 (− 0.018, 0.007)	− 0.006 (− 0.052, 0.040)	− 0.027 (− 0.050, − 0.005)*	− 0.010 (− 0.020, 0.0004)(*)
+ ethnicity	− 0.015 (− 0.030, − 0.0003)*	0.002 (− 0.010, 0.014)	0.013 (− 0.033, 0.059)	0.004 (− 0.014, 0.022)	− 0.005 (− 0.016, 0.005)
+ sex, age	− 0.013 (− 0.028, 0.001) (*)	0.006 (− 0.006, 0.018)	0.020 (− 0.026, 0.065)	0.001 (− 0.016, 0.019)	− 0.006 (− 0.016, 0.004)
+ comorbidities	− 0.015 (− 0.029, 0.0003) (*)	0.007 (− 0.005, 0.018)	0.022 (− 0.024, 0.067)	0.001 (− 0.017, 0.019)	− 0.006 (− 0.016, 0.004)
+ metabolic factors	− 0.016 (− 0.031, − 0.001)*	0.004 (− 0.005, 0.014)	0.020 (− 0.025, 0.064)	0.001 (− 0.017, 0.019)	− 0.006 (− 0.015, 0.002)
+ health behaviors	− 0.016 (− 0.031, − 0.001)*	0.005 (− 0.004, 0.015)	0.019 (− 0.026, 0.064)	0.0005 (− 0.017, 0.018)	− 0.006 (− 0.014, 0.003)
**Log S100B [µg/L]**	0.191 (0.128, 0.253)***	0.076 (0.031, 0.121)***	0.173 (0.007, 0.340)*	0.190 (0.111, 0.270)***	0.038 (0.001, 0.075)*
+ ethnicity	0.075 (0.019, 0.130)**	0.037 (− 0.009, 0.083)	0.077 (− 0.096, 0.250)	0.023 (− 0.044, 0.090)	0.012 (− 0.027, 0.051)
+ sex, age	0.063 (0.007, 0.119)*	0.006 (− 0.038, 0.050)	0.022 (− 0.151, 0.195)	0.047 (− 0.020, 0.114)	0.022 (− 0.017, 0.060)
+ comorbidities	0.061 (0.005, 0.118)*	0.011 (− 0.033, 0.054)	0.023 (− 0.151, 0.197)	0.047 (− 0.020, 0.114)	0.022 (− 0.017, 0.061)
+ metabolic factors	0.062 (0.006, 0.118)*	0.008 (− 0.029, 0.044)	0.020 (− 0.149, 0.190)	0.051 (− 0.015, 0.117)	0.027 (− 0.006, 0.059)
+ health behaviors	0.062 (0.006, 0.118)*	0.001 (− 0.035, 0.037)	0.015 (− 0.155, 0.185)	0.055 (− 0.011, 0.121)	0.031 (− 0.001, 0.063)(*)

BDNF, brain-derived neurotrophic factor; CLT, clot lysis time; GDNF, glia cell-line derived neurotrophic factor; PAI, plasminogen activator inhibitor; S100B, S100 calcium-binding protein B; VWF, von Willebrand factor. Unstandardized coefficients B (95% confidence interval) indicate associations between (log) neurotrophic and hemostatic factors with subsequent adjustment for blocks of covariates. Significance level: (*)*p* < 0.10, * *p* < 0.05, ** *p* < 0.01, *** *p* < 0.001.

Comorbidities: Human immunodeficiency virus positive status, history of atherothrombotic disease. Metabolic factors: Body mass index, hemoglobin A1c, total cholesterol to high-density lipoprotein cholesterol ratio; mean 24-h arterial pressure, estimated glomerular filtration rate, high-sensitivity C-reactive protein. Health behaviors: Current smoking, physical activity, excessive alcohol consumption.

### Stress reactivity

Across all participants, the Stroop provoked marked cardiovascular reactivity with increases (mean ± SD) in heart rate from 67 ± 11 bpm to 88 ± 16 bpm, in systolic BP from 138 ± 17 mmHg to 156 ± 21 mmHg, and in diastolic BP from 79 ± 10 mmHg to 89 ± 11 mmHg (all *p*-values < 0.001). Table 3 shows mean values of neurotrophic and hemostatic factors at baseline, after stress and as stress-induced percent changes after outliers had been removed. In all participants, there was a significant increase in VWF (*p* < 0.001) and a significant decrease in fibrinogen (*p* < 0.0.001), but no change in D-dimer (*p* = 0.87). Compared with whites, blacks showed a smaller increase in VWF as well as a greater decrease in fibrinogen from baseline to stress. Regarding neurotrophins, there was a significant decrease in both GDNF (*p* < 0.001) and S100B (*p* = 0.045) from baseline to stress in all participants. GDNF reactivity was significantly greater in whites, whereas S100B reactivity was significantly greater in blacks compared with their respective ethnic counterparts. After outlier values had been removed, median (range) for stress-induced percent changes were − 1.2% (− 67.1–58.3) for GDNF, 0.6% (− 21.0–21.3) for S100B, 4.0% (− 15.3–34.3) for VWF, − 6.9% (− 49.6–22.7) for fibrinogen and 0.7% (− 53.2–59.9) for D-dimer.

**Table 3:** Baseline values, values after stress and stress-induced percent changes in neurotrophins and hemostatic factors (without outliers).

Biological measures	All		Blacks		Whites		*p* (groups)
	Mean (95% CI)	n	Mean (95% CI)	n	Mean (95% CI)	n	
**Brain-derived neurotrophic factor**							
Baseline [ng/mL]	1.37 (1.31, 1.44)	408	1.25 (1.16,1.34)	200	1.51 (1.42, 1.60)	208	< 0.001
**Glia cell-line derived neurotrophic factor**							
Baseline [pg/mL]	50.8 (43.0, 59.7)	408	35.3 (28.9, 43.2)	200	71.9 (55.8, 92.7)	208	< 0.001
Stress [pg/mL]	43.2 (37.7, 49.5)	404	28.8 (24.3, 34.2)	197	63.5 (52.2, 77.3)	207	< 0.001
Change [%]	− 0.04 (− 2.09, 2.00)	404	− 2.73 (− 5.43, − 0.03)	197	2.52 (− 0.52, 5.55)	207	0.011
**S100 calcium-binding protein**							
Baseline [µg/L]	0.043 (0.041, 0.045)	406	0.050 (0.046, 0.054)	197	0.037 (0.035, 0.039)	209	< 0.001
Stress [µg/L]	0.042 (0.040, 0.043)	404	0.047 (0.044, 0.050)	196	0.038 (0.036, 0.039)	208	< 0.001
Change [%]	1.03 (0.43, 1.65)	403	2.25 (1.35, 3.14)	195	− 0.11 (− 0.89, 0.68)	208	< 0.001
**von Willebrand factor**							
Baseline [%]	74.1 (71.9, 76.4)	407	89.3 (86.1, 92.7)	198	62.1 (60.0, 64.2)	209	< 0.001
Stress [%]	92.2 (89.1, 95.5)	408	97.2 (93.7, 100.9)	199	87.6 (82.8, 92.8)	209	0.003
Change [%]	5.44 (4.48, 6.40)	409	2.14 (1.05, 3.24)	200	8.56 (7.14, 10.03)	209	< 0.001
**Fibrinogen**							
Baseline [g/L]	3.21 (3.14, 3.28)	409	3.41 (3.30, 3.53)	200	3.03 (2.95, 3.10)	209	< 0.001
Stress [g/L]	2.85 (2.79, 2.92)	408	2.94 (2.84, 3.04)	199	2.78 (2.69, 2.86)	209	0.014
Change [%]	− 9.66 (− 10.96, − 8.36)	404	− 11.62 (− 13.45, − 9.78)	197	− 7.79 (− 9.60, − 5.98)	207	0.004
**D-dimer**							
Baseline [ng/mL]	258 (239, 279)	402	303 (271, 339)	194	222 (200, 246)	208	< 0.001
Stress [ng/mL]	274 (250, 273)	402	330 (287, 378)	193	232 (205, 262)	209	< 0.001
Change [%]	1.20 (− 0.54, 2.93)	400	1.08 (− 1.51, 3.66)	194	1.31 (− 1.04, 3.66)	206	0.894
**Plasminogen activator inhibitor-1**							
Baseline [ng/mL]	26.4 (25.4, 27.4)	409	34.0 (32.6, 35.3)	200	20.7 (19.8, 21.6)	209	< 0.001
**Clot lysis time**							
Baseline [min]	79.0 (77.6, 80.4)	404	82.4 (80.2, 84.6)	195	76.0 (74.3, 77.7)	209	< 0.001

Values are geometric or percentage mean with 95% confidence interval (CI). See supplementary information for the number of outliers (i.e., 3 standard deviations above or below the sample mean). Two participants taking oral anticoagulants were additionally excluded for D-dimer measures.

### Neurotrophins at baseline and hemostatic factors in response to stress

**Figure 2:**
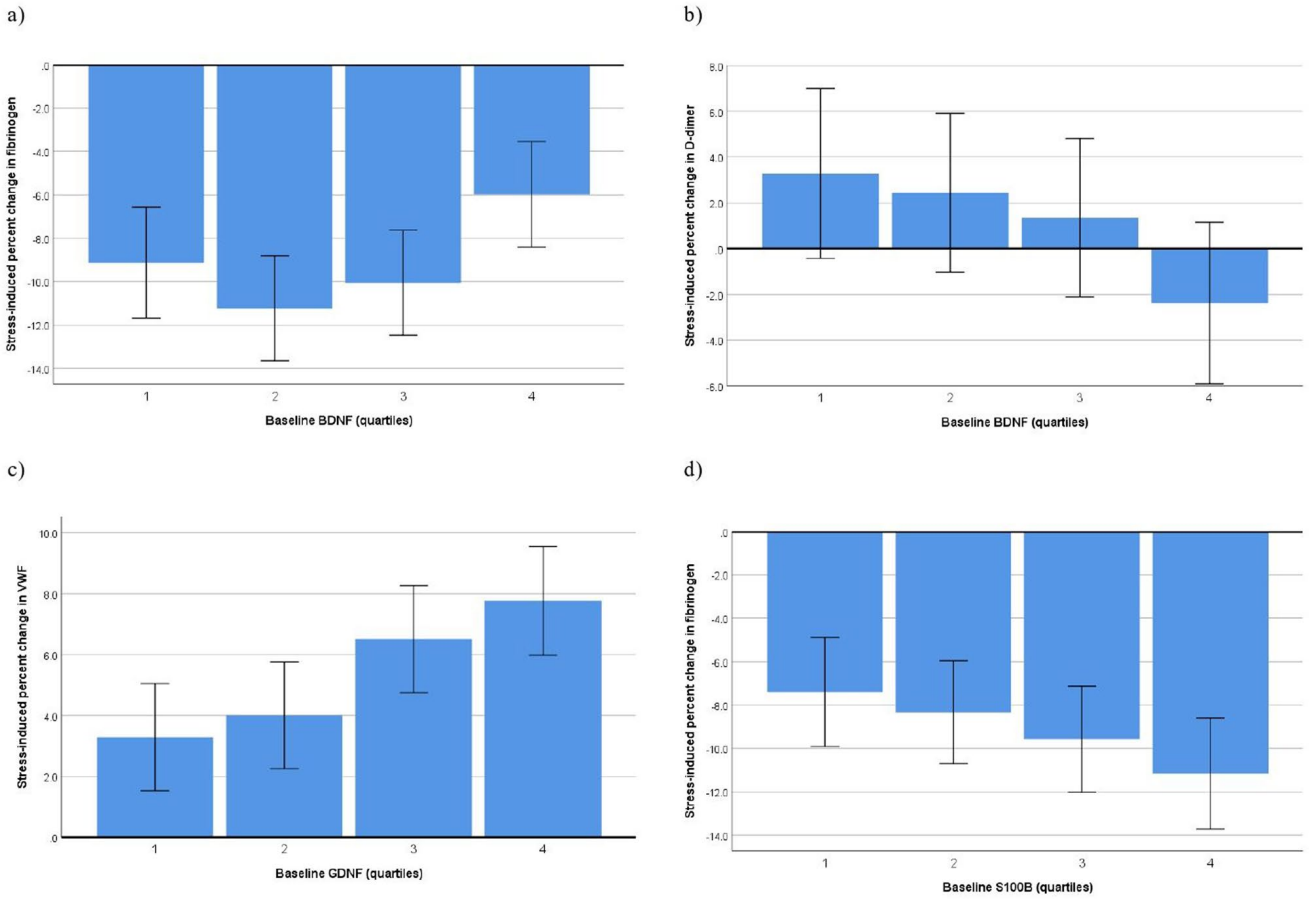
Fully adjusted significant associations between baseline values of neurotrophins across quartiles (1 = lowest quartile, 4 = uppermost quartile) and stress-induced percent changes in hemostatic factors. BDNF, brain-derived neurotrophic factor; GDNF, glial cell-derived neurotrophic factor; S100B, S100 calcium-binding protein; VWF, von Willebrand factor.

The regressions of baseline values of neurotrophins on stress-induced changes in hemostatic factors, subsequently adjusted for blocks of covariates, are summarized in Table 4. In fully adjusted models, higher baseline BDNF was significantly associated with less fibrinogen decrease (*p* = 0.003, d = 0.30) (Fig. 2a) and with lower D-dimer increase (*p* = 0.016, d = 0.25) (Fig. 2b) and, with borderline significance, with VWF (*p* < 0.09, d = 0.18). Higher baseline GDNF was significantly associated with greater VWF increase (*p* = 0.001, d = 0.35) (Fig. 2c). Greater baseline S100B was significantly associated with greater fibrinogen decrease (*p* = 0.048, d = 0.20) (Fig. 2d) and, with borderline significance, with greater VWF increase in the unadjusted analysis (*p* = 0.002, d = 0.32). In complementary analyses, neurotrophins showed no significant interactions with ethnicity (data not shown).

**Table 4:** Regression models for the associations between neurotrophins at baseline and stress-induced changes in hemostatic factors.

Variables entered	VWF stress change [%]	Fibrinogen stress change [%]	D-dimer stress change [%]
**Log BDNF [ng/mL]**	− 3.719 (− 8.258, 0.819)	8.781 (3.106, 14.456)**	− 8.872 (− 16.986, − 0.757)*
+ Ethnicity	− 6.995 (− 11.335, − 2.654)**	7.353 (1.585, 13.120)*	− 9.325 (− 17.607, − 1.044)*
+ Sex, age	− 4.296 (− 8.803, 0.211)(*)	8.836 (2.784, 14.889)**	− 11.600 (− 20.340, − 2.860)**
+ Comorbidities	− 4.293 (− 8.818, 0.232)(*)	9.028 (2.968, 15.088)**	− 11.298 (− 20.065, − 2.530)*
+ Metabolic factors	− 4.323 (− 8.875, 0.230)(*)	9.164 (3.107, 15.221)**	− 10.528 (− 19.212, − 1.845)*
+ Health behaviors	− 3.975 (− 8.512, 0.561)(*)	9.181 (3.098, 15.263)**	− 10.684 (− 19.377, − 1.992)*
**Log GDNF [pg/ml]**	3.033 (1.771, 4.296)***	0.523 (− 1.133, 2.179)	− 0.875 (− 3.235, 1.485)
+ Ethnicity	2.234 (0.997, 3.472)***	0.032 (− 1.643, 1.708)	− 0.949 (− 3.366, 1.468)
+ Sex, age	1.999 (0.784, 3.214)**	0.050 (− 1.630, 1.730)	− 0.854 (− 3.284, 1.577)
+ Comorbidities	2.007 (0.784, 3.230)**	0.052 (− 1.743, 1.639)	− 0.849 (− 3.282, 1.584)
+ Metabolic factors	2.092 (0.858, 3.326)***	0.051 (− 1.647, 1.750)	− 0.786 (− 3.202, 1.630)
+ Health behaviors	2.169 (0.941, 3.398)***	0.062 (− 1.645, 1.770)	− 0.909 (− 3.330, 1.512)
**Log S100B [µg/L]**	− 7.455 (− 12.124, − 2.785)**	− 8.627 (− 14.553, − 2.701)**	3.603 (− 4.870, 12.076)
+ Ethnicity	− 2.674 (− 7.396, 2.047)	− 6.409 (− 12.632, − 0.186)*	3.809 (− 5.123, 12.740)
+ Sex, age	− 0.600 (− 5.294, 4.095)	− 6.636 (− 12.955, − 0.317)*	3.054 (− 6.069, 12.176)
+ Comorbidities	− 0.629 (− 5.346, 4.098)	− 6.976 (− 13.313, − 0.639)*	3.341 (− 5.780, 12.462)
+ Metabolic factors	− 0.464 (− 5.204, 4.276)	− 6.593 (− 12.919, − 0.267)*	3.182 (− 5.820, 12.184)
+ Health behaviors	− 0.701 (− 5.433, 4.031)	− 6.419 (− 12.792, − 0.046)*	3.310 (− 5.736, 12.356)

BDNF, brain-derived neurotrophic factor; GDNF, glia cell-line derived neurotrophic factor; S100B, S100 calcium-binding protein B; VWF, von Willebrand factor.

Unstandardized coefficients B (95% confidence interval) indicate the percent change from baseline in hemostatic factors with a 1-unit increase in (log) baseline levels of neurotrophins, subsequently adjusted for blocks of covariates. Significance level: (*)*p* < 0.10, * *p* < 0.05, ** *p* < 0.01, *** *p* < 0.001.

Comorbidities: Human immunodeficiency virus positive status, history of atherothrombotic disease. Metabolic factors: Body mass index, hemoglobin A1c, total cholesterol to high-density lipoprotein cholesterol ratio; mean 24-h arterial pressure, estimated glomerular filtration rate, high-sensitivity C-reactive protein. Health behaviors: Current smoking, physical activity, excessive alcohol consumption.

### Neurotrophic and hemostatic factors in response to stress

**Figure 3:**
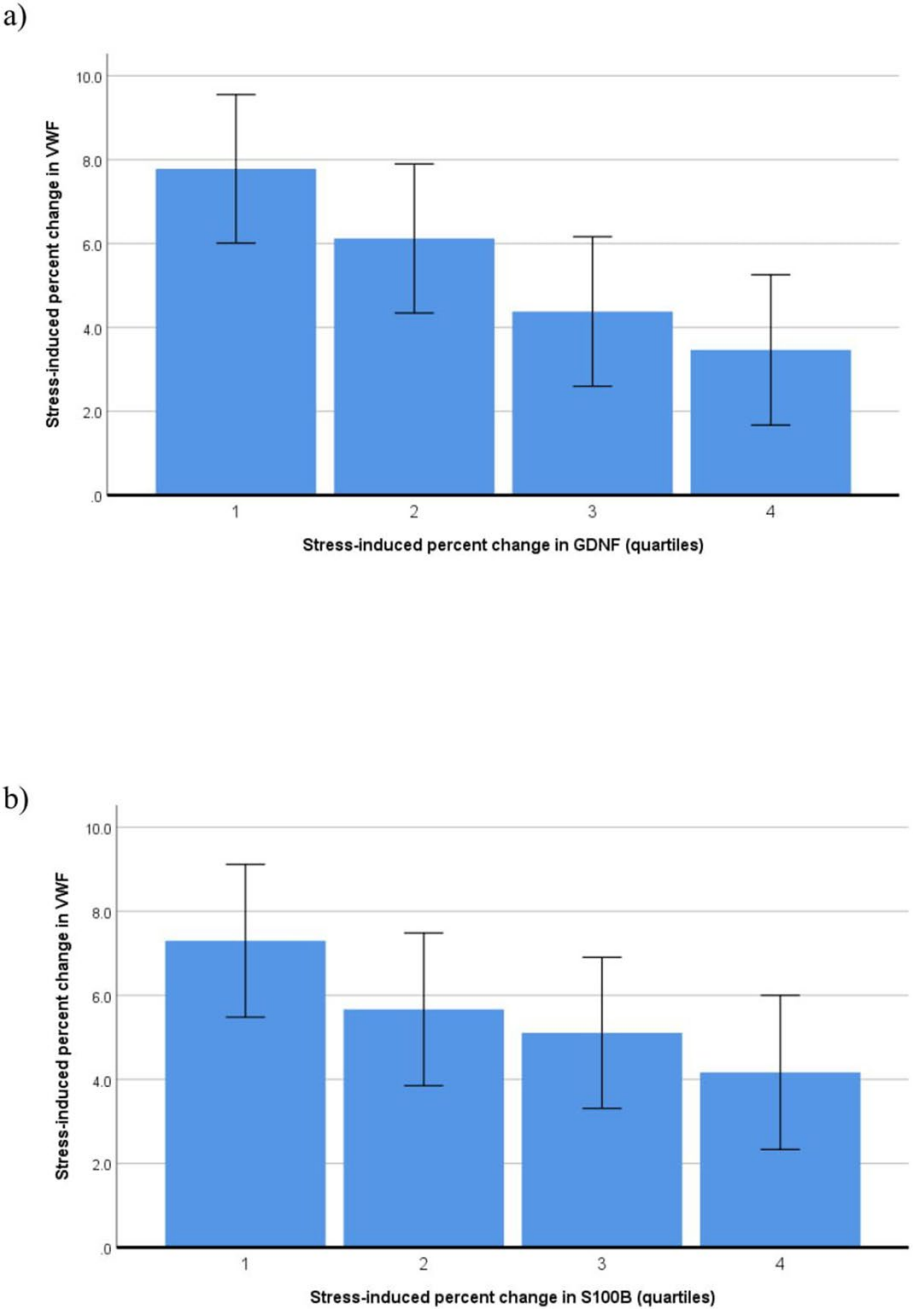
Fully adjusted significant associations between stress-induced percent changes in neurotrophins across quartiles (1 = lowest quartile, 4 = uppermost quartile) and stress-induced percent changes in von Willebrand factor (VWF). GDNF, glial cell-derived neurotrophic factor; S100B, S100 calcium-binding protein.

The regressions of stress-induced changes in neurotrophins on stress-induced changes in hemostatic factors, subsequently adjusted for blocks of covariates, are summarized in Table 5. Greater increases in both GDNF (*p* = 0.006, d = 0.28) (Fig. 3a) and S100B (*p* = 0.042, d = 0.21) (Fig. 3b) were significantly associated with lower VWF increase in fully adjusted models. In the unadjusted analysis, there also was a trend towards statistical significance for an association of greater S100B increase with lower fibrinogen increase (*p* < 0.09, d = 0.17). No significant associations were observed between stress changes in neurotrophins and D-dimer.

**Table 5:** Regression models for the associations between stress-induced changes in neurotrophins and hemostatic factors.

Variables entered	VWF stress change [%]	Fibrinogen stress change [%]	D-dimer stress change [%]
**GDNF stress change [%]**	− 0.044 (− 0.090, 0.003)(*)	0.011 (− 0.048, 0.069)	0.035 (− 0.049, 0.118)
+ ethnicity	− 0.064 (− 0.108, − 0.020)**	− 0.0003 (− 0.059, 0.058)	0.034 (− 0.050, 0.118)
+ sex, age	− 0.059 (− 0.102, − 0.016)**	− 0.004 (− 0.063, 0.055)	0.032 (− 0.053, 0.117)
+ comorbidities	− 0.059 (− 0.102, − 0.016)**	− 0.004 (− 0.062, 0.055)	0.029 (− 0.056, 0.114)
+ metabolic factors	− 0.060 (− 0.103, − 0.017)**	− 0.005 (− 0.064, 0.053)	0.024 (− 0.060, 0.108)
+ health behaviors	− 0.060 (− 0.103, − 0.017)**	− 0.006 (− 0.065, 0.052)	0.026 (− 0.058, 0.110)
**S100B stress change [%]**	− 0.292 (− 0.447, − 0.137)***	− 0.173 (− 0.372, 0.026)(*)	0.049 (− 0.238, 0.336)
+ ethnicity	− 0.200 (− 0.352, − 0.049)**	− 0.123 (− 0.324, 0.078)	0.055 (− 0.237, 0.348)
+ sex, age	− 0.166 (− 0.315, − 0.018)*	− 0.129 (− 0.331, 0.072)	0.041 (− 0.253, 0.335)
+ comorbidities	− 0.166 (− 0.315, − 0.016)*	− 0.124 (− 0.327, 0.078)	0.045 (− 0.250, 0.339)
+ metabolic factors	− 0.155 (− 0.307, − 0.004)*	− 0.100 (− 0.304, 0.105)	− 0.004 (− 0.299, 0.290)
+ health behaviors	− 0.157 (− 0.308, − 0.006)*	− 0.102 (− 0.308, 0.103)	0.017 (− 0.278, 0.311)

GDNF, glia cell-line derived neurotrophic factor; S100B, S100 calcium-binding protein B; VWF, von Willebrand factor.

Unstandardized beta-coefficients B (95% confidence interval) indicate the percent change in hemostatic factors from baseline with a 1% increase in neurotrophins from baseline, subsequently adjusted for blocks of covariates. Significance level: (*)*p* < 0.10, * *p* < 0.05, ** *p* < 0.01, *** *p* < 0.001.

Comorbidities: Human immunodeficiency virus positive status, history of atherothrombotic disease. Metabolic factors: Body mass index, hemoglobin A1c, total cholesterol to high-density lipoprotein cholesterol ratio; mean 24-h arterial pressure, estimated glomerular filtration rate, high-sensitivity C-reactive protein. Health behaviors: Current smoking, physical activity, excessive alcohol consumption.

**Figure 4:**
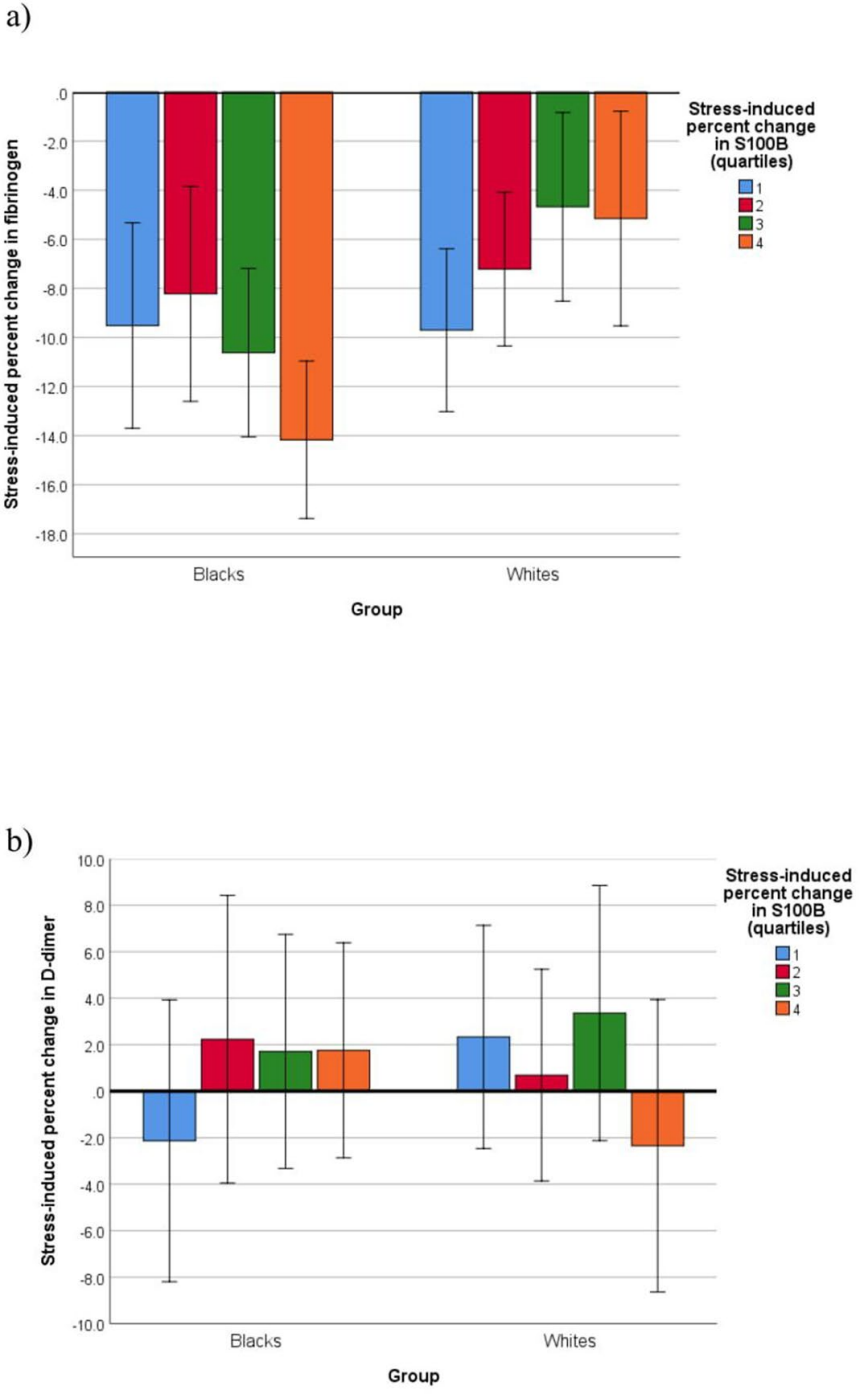
Fully adjusted significant association interactions between ethnicity and stress-induced percent change in S100 calcium-binding protein (S100B) across quartiles (1 = lowest quartile, 4 = uppermost quartile) for stress-induced percent changes in hemostatic factors.

In fully adjusted complementary analyses, there emerged significant interactions between ethnicity and stress-induced changes in S100B for changes in fibrinogen (*p* = 0.041, d = 0.21) and D-dimer (*p* = 0.045, d = 0.21). Follow-up analyses revealed an inverse association between stress-induced changes in S100B and fibrinogen in blacks (B = − 0.315, 95% CI − 0.586, − 0.043; *p* = 0.023, d = 0.35), but a positive association in whites (B = 0.070, 95% CI − 0.254, 0.393; *p* = 0.67). That is, a greater stress-induced change in S100B was associated with a greater decrease in fibrinogen in blacks but a smaller decrease in whites (Fig. 4a). Stress-induced changes in S100B and D-dimer showed an inverse association in whites (B = − 0.287, 95% CI − 0.721, 0.147; *p* = 0.19, d = 0.19) and a positive association in blacks (B = 0.238, 95% CI − 0.167, 0.643; *p* = 0.25, d = 0.18). That is, a stress-induced increase in S100B was associated with a D-dimer increase in blacks, but with a decrease in whites (Fig. 4b).

## Discussion

In a cohort of black and white South Africans, we found several significant relationships between neurotrophic and hemostatic measures both at baseline and during acute mental stress, showing small-to-moderate effects, suggesting clinical relevance. Associations with a trend towards statistical significance with close to small effect sizes complemented the pattern of relationships. The findings of our study may support the existence of a peripheral neurotrophin-hemostasis interaction.

Regarding relationships at baseline, we found a significant association of lower GDNF on the one hand and of higher S100B on the other with higher VWF levels, both in the unadjusted and fully adjusted analyses (Fig. 1a,b), suggesting a robust association. Moreover, in the unadjusted analysis, but with borderline significance only in the fully adjusted model, higher S100B was associated with longer CLT indicative of reduced fibrinolytic activity. S100B is a biomarker of cerebral injury, neuroinflammation and blood brain barrier permeability^4^, and S100B was also found to be increased in patients with ACS^5^; therefore, it is conceivable that the higher the circulating S100B levels, the greater the procoagulant tendency at baseline could be. Corroborating a previous study in trauma patients showing a direct relationship between S100B and VWF^17^ and knowing that S100B may stimulate endothelial cells^39^ secreting VWF^16^, endothelial cell function could link S100B with VWF. As GDNF ensures survival of neurons^40^, elevated levels should be associated with better health across many diseases, mirrored by the fact that high GDNF levels were associated with low VWF in our study. Again, endothelium-mediated mechanisms could be involved, as GDNF enhances tight junctions of endothelial cells of the blood brain barrier^41^. Also, GDNF induces the differentiation of amniotic fluid-derived stem cells into vascular endothelial-like cells positive for VWF^42^. However, the exact mechanisms through which S100B and GDNF affect hemostatic function in the periphery remain to be determined. Further direct associations of baseline S100B with fibrinogen, D-dimer, and PAI-1, and to a lesser extent of lower baseline GDNF with markers indicating impaired fibrinolysis, were explained by ethnicity. BDNF showed several associations with hemostatic factors, even when controlling for ethnicity; however, significance was lost after adjusting for sex and age. Overall, these results suggest that demographic factors are to be accounted for when studying the role of neurotrophins in baseline hemostatic function.

To study relationships between neurotrophic and hemostatic factors during sympathetic challenge, we applied a standard laboratory stress. Activation of the sympathetic nervous system was evidenced by marked increases in hemodynamic parameters. As previously reported in this sample^27^, acute mental stress also provoked a significant increase in VWF between baseline and 10 min post-stress, whereas during this time interval fibrinogen decreased and D-dimer did not change. The increase in VWF concurs with abundant research showing that VWF can be acutely released from Weibel-Palade bodies in endothelial cells, such as through a stress-induced catecholamine surge^22^. In contrast, the decrease in fibrinogen levels was unexpected, because acute mental stress provoked increased plasma fibrinogen concentrations in numerous previous studies as reviewed elsewhere^22,43,44^. One explanation could be that a one-minute stress task was too short to induce a significant increase in fibrinogen, and perhaps also hemoconcentration. A hemodynamically-induced plasma shift into extravascular spaces is one mechanism that may underlie an increase in the plasma fibrinogen concentration during acute mental stress^45^. Another explanation could be that speech stress, conferring social evaluative threat^46^, or the combination of the Stroop with another stress like mental arithmetic^31^ have greater potency in provoking significant fibrinogen responses than the Stroop alone^47^. A circadian decrease during the morning hours might also explain reduced fibrinogen concentration, although this effect should be minimal within an 11-min period between baseline and stress sampling of fibrinogen measures. Explanations similar to those for the fibrinogen results could explain the lack of a stress-induced increase in D-dimer levels, including a minor plasma volume shift^45^ with a one-minute stress task, and the characteristics of the stress task. D-dimer increase has particularly been shown in studies applying speech stressors^46,48^, but not for instance when combing the Stroop with mental arithmetic^49^. The varying sensitivity of the different D-dimer assays used in previous stress studies may be another explanation for heterogeneous study results.

Interestingly, across all study participants, we observed a significant decrease in both GDNF and S100B, the latter opposite to the effects of restraint stress in rats^23^. However, S100B dynamics could vary between studies, as in the rat model S100B was measured two hours after stress, whereas in our study S100B was measured 10 min post-stress. To our knowledge, no studies to date have examined circulating levels of GDNF pre- and post-stress applying a standardized stress paradigm, so more research is needed to pinpoint with greater confidence not only the stress-responsiveness of GDNF but also of S100B in humans along with the involved mechanisms.

In partial agreement with our pre-specified hypothesis, lower baseline levels of BDNF were associated, on the one hand, with significantly greater stress-induced increases in D-dimer (Fig. 2b) and, with borderline significance, in VWF. Contrary to our hypothesis, there was a significantly greater decrease in fibrinogen with lower BDNF levels at baseline (Fig. 2a). However, as previously discussed, decreased fibrinogen levels could also indicate a dysfunctional stress response in our sample^27^. To concur, in a previous study with healthy adults, smaller acute mental stress-induced fibrinogen responses were associated with a greater chance of detecting plasma cardiac troponin T, indicating a disadvantage for cardiovascular health^50^. Therefore, the observed pattern of associations could tentatively indicate that lower BDNF levels are associated with hemostatic changes posing a threat rather than a physiologic advantage to the cardiovascular system during sympathetic activation^20^.

Unfortunately, as BDNF will likely increase during sympathetic bursts^23^, we did not assess stress responses in BDNF, which might have yielded a different pattern of associations. To support such an argument, we found lower baseline levels of GDNF (Fig. 2c), but greater stress-induced increase in GDNF (Fig. 3a), both to be significantly associated with smaller stress-induced increase in VWF. Thus, a GDNF profile of low baseline/high reactivity appears to be associated with low VWF response. Nonetheless, this finding could also be explained by the law of initial values stating that those with low baseline values GDNF are likely to demonstrate the largest increases. We further found that greater baseline levels of S100B was associated with greater fibrinogen decrease (Fig. 2d) and greater stress-induced increase in S100B was associated with smaller VWF responses (Fig. 3b). The significant associations that emerged during sympathetic challenge were largely unaffected by covariates. The association between GDNF and VWF during stress could relate to sympathetic nervous system function. GDNF has been shown to guide sympathetic nerves to the heart^51^, and VWF is arguably one of the most responsive molecules to sympathetic challenges^22^. In cultured astrocytes, S100B induces generation of nitric oxide^52^ and endogenously produced NO may dampen the regulated pathway of VWF secretion^53^, further underscoring a role of endothelial cells.

As published earlier^25–27^, or to be published in more detail elsewhere, there were ethnic differences in hemostatic factors and neurotrophins both in terms of baseline concentrations and stress reactivity. Here, we were particularly interested in ethnicity as a potential moderating variable in the neurotrophin-hemostasis association. We found that the effect of ethnicity was confined to associations between stress-induced changes in both neurotrophic and hemostatic factors. Greater stress-induced increase in S100B was associated with a greater fibrinogen decrease in blacks than whites (Fig. 4a), and with an increase in D-dimer in blacks, whereas whites showed a decrease (Fig. 4b). These associations do not allow a straightforward interpretation in terms of increased cardiovascular harm in blacks, above and beyond their apparently unfavorable metabolic and hemostatic risk profile.

The novelty of the investigated associations, a comparably large and diverse sample, taking into account important covariates of hemostatic function, and application of a standardized stress paradigm are strengths of our study, which also has its limitations. As discussed above, the short-term stress task could have prevented full reactivity of neurotrophins and hemostatic factors. Also, the interval between blood sampling before and after stress was only 11 min which could have been too short for biological measures to reach peak responses. Hemostatic factors like fibrinogen and D-dimer show significant time-dependent intra-individual variation^54^. Diurnal variation has also been shown in BDNF^55^, but not in S100B^56^. Unfortunately, we did not include a non-stress control group, so we are unable to exclude potential time-dependent effects on biological measures, including diurnal ones. We did not have information on hematocrit and hemoglobin before and after stress to calculate stress-induced plasma volume concentration. Arithmetic adjustment of the concentration of hemostatic factors could have provided more insight into the mechanisms of the observed relationships between neurotrophins and hemostatic factors during stress^45^. The cross-sectional study design precludes causal inferences, such that changes in hemostatic factors could also result in changes of neurotrophin levels. Residual confounding by third unknown factors affecting both brain and hemostatic function cannot be excluded. This includes the ABO blood group for which we had no information in our study. Individuals with blood group O have an approximately 25% lower plasma VWF concentration. In the South African population, the frequency of blood group O is 45%^57^. However, to what extent blood group O may influence the stress-induced reactivity of VWF is currently unknown. Blacks differed in many health characteristics from whites, including that 9.5% of our black study participants were HIV-positive, but we controlled in our analyses for these differences. Nevertheless, our findings from a population subgroup in South Africa should not be generalized to populations with a different cardiovascular risk profile and ethnic composition. A range of additional metabolic factors could additionally be adjusted for, e.g. glucose, insulin or insulin resistance, but with the risk of overadjustment of regression models and multicolinearity. Whether circulating levels of neurotrophic factors reflect glial function or also originate considerably from extracranial sources, such as the adipose tissue in the case of S100B^58^ or platelets in the case of BDNF^59^, remains to be clarified. Platelet activity and BDNF stress reactivity were not assessed. Also, we did not perform global coagulation assays like the activated partial thromboplastin time or prothrombin time, which would have helped to better integrate the putative functional consequences of observed variations in VWF, fibrinogen and D-dimer concentrations on coagulability. Although effect sizes suggested clinically relevant associations, we do not know the clinical implications of a peripheral neurotrophin-hemostasis link in terms of an increased cardiovascular risk.

To conclude, the observed associations between neurotrophic and hemostatic factors in the peripheral circulation, independent of demographics, metabolic factors and health behaviors, supports a relevant role of a brain–heart link. The potential implications of this interaction for atherothrombotic cardiovascular disease need to be explored further, particularly as the effects were in the small-to-moderate range of clinical relevance.

## Supplementary Information

Supplementary Information.

Supplementary Information.

## Data Availability

Deidentified individual participant data and the data analysis plan are available from the corresponding author on reasonable request.
