# Supplementary material for: Circulating neurotrophins and hemostatic risk factors of atherothrombotic cardiovascular disease at baseline and during sympathetic challenge: the SABPA study
Source: Sci Rep. 2021 Jan 27;11:2297. doi: 10.1038/s41598-021-81946-6 (PMC7841151; doi:10.1038/s41598-021-81946-6)
Supplement: Supplementary file 1 — Supplementary Information. [file 41598_2021_81946_MOESM1_ESM.docx]

**Circulating neurotrophins and hemostatic risk factors of atherothrombotic cardiovascular disease at baseline and during sympathetic challenge: The SABPA study**

^1,2^ Roland von Känel, MD

^3^Mark Hamer, PhD

^2^Annemarie Wentzel, PhD

^2^Leoné Malan, RN, PhD

^1^ Department of Consultation-Liaison Psychiatry and Psychosomatic Medicine, University Hospital Zurich, University of Zurich, Zurich, Switzerland

^2^ Hypertension in Africa Research Team (HART), North-West University, Potchefstroom, South Africa

^3^ Division of Surgery & Interventional Science, Faculty of Medical Sciences, University College London, United Kingdom
