# Supplementary material for: Circulating neurotrophins and hemostatic risk factors of atherothrombotic cardiovascular disease at baseline and during sympathetic challenge: the SABPA study
Source: Sci Rep. 2021 Jan 27;11:2297. doi: 10.1038/s41598-021-81946-6 (PMC7841151; doi:10.1038/s41598-021-81946-6)
Supplement: Supplementary file 2 — Supplementary Information. [file 41598_2021_81946_MOESM2_ESM.docx]

**Supplementary information**

**Numbers of missing values**

There were several variables with missing values, which were replaced with the expectation maximization algorithm. Data imputation was made in 2 cases each for GGT, CRP, T-C, HDL-C, PAI-1 and BDNF; in 3 cases for baseline S100B; in 4 cases for eGFR; in 6 cases each for HbA1c, baseline D-dimer, baseline HR, baseline systolic BP and baseline diastolic BP; in 8 cases for post-stress GDNF; in 9 cases for baseline VWF; in 10 cases each for baseline fibrinogen and post-stress S100B; in 12 cases each for post-stress VWF, HR during stress, systolic BP during stress, diastolic BP during stress; in 13 cases for post-stress fibrinogen; in 15 cases for post-stress D-dimer; and in 17 cases for CLT.

**Numbers of outliers**

Log baseline values, log post-stress values, and stress-induced percent changes of neurotrophic and hemostatic factors that were 3 SDs above or below the sample mean were considered outliers. This applied to baseline BDNF and GDNF measures in 1 case each, baseline VWF measures in 2 cases, baseline S100B measures in 3 cases, and baseline D-dimer and CLT measures in 5 cases each. Outliers for post-stress values were present in 1 case each for VWF and fibrinogen measures and in 5 cases each for D-dimer, GDNF, and S100B measures. Outliers of stress-induced change scores were observed in 5 cases each for fibrinogen and GDNF, in 6 cases for S100B, and in 7 cases for D-dimer.
